# Circ_0047921 acts as the sponge of miR-1287-5p to stimulate lung cancer progression by regulating proliferation, migration, invasion, and glycolysis of lung cancer cells

**DOI:** 10.1186/s12957-021-02466-1

**Published:** 2022-04-02

**Authors:** Yuehua Xiao, Shequn Gu, Wenxiu Yao, Ling Qin, Jihui Luo

**Affiliations:** 1 The First General Surgery Department of Carcinoma, The First People’s Hospital of Chenzhou, Southern Medical University, Chenzhou, Hunan China; 2 The First Department of Medical Oncology, The First People’s Hospital of Chenzhou, Southern Medical University, Chenzhou, Hunan China; 3 Department of Thoracic Oncology, The Cancer Hospital of Sichuan Province, Chengdu, Sichuan China; 4 The Second General Surgery Department of Carcinoma, The First People’s Hospital of Chenzhou, Southern Medical University, Chenzhou, 423000 Hunan China

**Keywords:** Circ_0047921, MiR-1287-5p, LARP1, Lung cancer

## Abstract

**Background:**

Lung cancer is a common respiratory system disease caused by multiple factors. Circular RNAs (circRNAs) play vital roles in tumorigenesis, including lung cancer. This study aimed to clarify the role and underlying molecular mechanisms of circ_0047921 in lung cancer.

**Methods:**

Real-time quantitative polymerase chain reaction (RT-qPCR) was used to assess the expression levels of circ_0047921, La-related protein 1 (LARP1), and miR-1287-5p. Cell proliferation was analyzed by CCK-8 and EdU assays. Transwell assay was used to assess migration and invasion. Western blot assay was employed to quantify protein expression. Glycolysis ability of cell was determined by measuring glucose consumption and lactate production with matched kits. The relationship between miR-1287-5p and circ_0047921 or LARP1 was confirmed by dual-luciferase reporter assay. In addition, a xenograft model was established to clarify the functional role of circ_0047921 in vivo.

**Results:**

Circ_0047921 was highly expressed in lung cancer tissues and cells. Circ_0047921 downregulation repressed proliferation, migration, invasion, epithelial-mesenchymal transition (EMT) and glycolysis in lung cancer cells. Circ_0047921 targeted miR-1287-5p to deplete miR-1287-5p expression. The effects caused by circ_0047921 downregulation were reversed by miR-1287-5p inhibition. In addition, LARP1 was a target of miR-1287-5p, and circ_0047921 could directly interact with miR-1287-5p to increase the expression of LARP1. The effects caused by circ_0047921 downregulation were also reversed by LARP1 overexpression. Circ_0047921 silencing impeded the growth of tumor in vivo.

**Conclusion:**

Circ_0047921 was overexpressed in lung cancer, and circ_0047921 targeted miR-1287-5p to modulate LARP1 expression, thereby facilitating the development of lung cancer.

**Trial registration:**

The present study was approved by the ethical review committee of The First People’s Hospital of Chenzhou, Southern Medical University with reference no. 20210106.

## Highlights

1. Circ_0047921 was upregulated in lung cancer tissues and cells.

2. Circ_0047921/miR-1287-5p/LARP1 axis played a key role in proliferation, migration, invasion, EMT and glycolysis of lung cancer cells.

3. Circ_0047921 regulated LARP1 by sponging miR-1287-5p in lung cancer.

## Introduction

Lung cancer is the foremost cause of cancer-related death of both men and women all over the world [1]. Studies state that smoking results in about two-thirds of lung cancer deaths worldwide, and other factors, such as PM2.5 air pollution (due to industrial waste gas, automobile exhaust and etc.) and inhalable agents (burning of solid fuels), also induce the incidence of lung cancer [1], which also elevates health expenditure and socioeconomic burden [2, 3]. Local invasion and distant metastases are considered to be main risk factors to poor clinical results of patients with lung cancer [4]. Given that the detailed molecular characterization of lung cancer has not been fully elucidated, it is imperative to illuminate the pathogenesis and develop personalized therapeutic strategies for lung cancer.

Recently, evidence has shown that circular RNAs (circRNAs) may be involved in lung cancer pathogenesis, providing novel insights into disease biology [5]. CircRNAs are a special type of regulatory noncoding RNAs that are formed through an alternative splicing mechanism [6]. CircRNAs have numerous biologic functions, including controlling development, acting regulatory role in gene expression, and participating in oncogenesis [7, 8]. For example, circ_100395 was shown to participate in lung cancer through modulating cell proliferation, migration and invasion [9]. Whereas, most circRNAs have not been functionally explored in biological processes. Circ_0047921 is derived from exon4 of CD266 gene and locates on chr18 (67592936-67563281). However, the function of circ_0047921 remains unclear in lung cancer up to date.

Numerous researches have shown that circRNAs can act as competitive endogenous RNAs (ceRNAs) by sponging miRNAs [10]. MiR-1287-5p functioned as a tumor inhibitor in various cancers, including breast cancer [11], colorectal cancer [12], and pancreatic carcinoma [13]. In non-small cell lung cancer (NSCLC), miR-1287 was proved to be sponged by circ_0016760 and circ_0026134 [14, 15]. Nevertheless, there are no relevant studies on the relationship between miR-1287-5p and circ_0047921.

La-related protein 1 (LARP1), conserved RNA-binding protein, exerts its cancerogenic functions via mediating multiple malignant behavior in cancer cells [16, 17]. For example, Mura et al. confirmed that LARP1 promoted cell growth, mobility and tumor formation [16]. Moreover, overexpression of LARP1 predicted poor prognosis in colorectal cancer [18]. In lung cancer, silencing of LARP1 constrained growth and mobility of cancer cells, indicating LARP1 was an oncogene [19]. However, whether LARP1 can be regulated by miR-1287-5p needs further elucidation.

The present work ensured the expression of circ_0047921 in lung cancer and investigated its function on proliferation, mobility, epithelial-mesenchymal transition (EMT) and glycolysis in lung cancer cells. Moreover, we explored the interplays among circ_0047921, miR-1287-5p, and LARP1, so as to provide new biomarkers for lung cancer diagnosis or treatment.

## Materials and methods

### Patient specimens

Lung cancer tissues (NSCLC) and neighboring non-tumor tissues were harvested from patients (*n* = 60) at The First People’s Hospital of Chenzhou, Southern Medical University. The written informed consents were offered by patients. All removed tissues were frozen in liquid nitrogen and then transferred to – 80 °C for subsequent study. This study was premised by the Ethics Committee of The First People’s Hospital of Chenzhou, Southern Medical University.

### Cell lines and cell culture

Human bronchiolar epithelial cell line (BEAS-2B) and lung cancer cell lines (A549, H1299, H1650, Calu3, and SK-MES-1) were gained from the Chinese Academy of Sciences (Shanghai, China). All cells were maintained in RPMI 1640 medium (Wisent, Shanghai, China) supplemented with 10% (v/v) fetal bovine serum (FBS; Biochrom KG, Berlin, Germany) under standard culture condition (5% CO_2_, 37 °C). In addition, 1% penicillin/streptomycin (Solarbio, Beijing, China) was added into medium to prevent bacterial contamination.

### Real-time quantitative polymerase chain reaction (RT-qPCR)

Total RNA was isolated using Trizol reagent (Thermo Fisher Scientific, Waltham, MA, USA) in accordance with the user’s guideline. For mRNA and lncRNA quantification, complementary DNA was synthesized using SuperScript Reverse Transcriptase Kit (Vazyme, Nanjing, China). The transcript levels of circ_0047921 and LARP1 were evaluated by performing RT-qPCR assay on Applied Biosystems 7500 Fast Real-Time PCR System (Applied Biosystems, Foster City, CA, USA) based on the 2^−ΔΔCt^ method, with β-actin as internal control. For miRNA quantification, microRNA Reverse Transcription Kit (Qiagen, Hilden, Germany) was used for reverse transcription, and U6 served as an internal control. Three wells were set for one sample in one experiment, and a total of three independent experiments were performed.

The primers were synthesized by Sangon Biotech (Shanghai, China) and sequences of primers were listed as follows:

- circ_0047921 (F, 5′-GCAGGCCAGCGCAGGA-3′; R, 5′-CAGGTTCCGAAACAATGT-3′);
- miR-1287-5p (F, 5′-GCCGAGTGCTGGATCAGTGG-3′; R, 5′-CTCAACTGGTGTCGTGGA-3′);
- LARP1 (F, 5′-GCTGTTTAGGAACAGCTGCC-3′; R, 5′-CCACAGGTGACAGGGAGAAG-3′);
- β-actin (F, 5′-GCCGGGACCTGACTGACTAC-3′; R, 5′-TCTCCTTAATGTCACGCACGAT-3′); U6 (F, 5′-AACGCTTCACGAATTTGCGT-3′; R, 5′-CTCGCTTCGGCAGCACA-3′).

### Transfection assay

Small interfering RNA (siRNA) against circ_0047921 (si-circ_0047921), siRNA scrambled control (si-NC), LARP1-overexpression vector, and control pcDNA were designed by Genscript (Nanjing, China). MiR-1287-5p mimic (miR-1287-5p), mimic negative control (miR-NC), miR-1287-5p inhibitor (miR-1287-5p inhibitor), and inhibitor negative control (inhibitor-NC) were purchased from Sangon Biotech. Lung cancer cells (1 × 10^5^) were inoculated into 6-well plates and transfected with above oligonucleotides or vectors using Lipofectamine 2000 (Thermo Fisher Scientific) in accordance with the producer’s direction. Cells were collected for further analyses at 48 h post-transfection and transfection efficacy was investigated by RT-qPCR assay.

### Cell counting kit-8 (CCK8) assay

Cell viability was examined using CCK8 assay referring to the user’s guideline. Briefly, approximately 2 × 10^3^ cells/well were inoculated into 96-well plates. After incubation for indicated time points, 10 μL of CCK8 (Vazyme) was added into each well and incubated for another 1 h at 37 °C. Cell viability was assessed by measuring 450 nm absorbance at each indicated time point under microplate reader (Aolu Biotech, Shanghai, China). Each contained three replicate wells, and three independent experiments were conducted.

### 5-ethynyl-2′-deoxyuridine (EdU) assay

The 5-ethynyl-2′-deoxyuridine (EdU) assay kit was purchased from RiboBio (Nanjing, China) for proliferation measurement as previous description [20]. After transfection for 48 h, 50 μM of EdU was added into A549 and H1299 cells and then indicated with 1.5 h, and DAPI was utilized to stain nucleus. The EdU-positive cells were determined under the using the fluorescence microscope (Thermo Fisher Scientific). Three independent experiments were conducted.

### Migration and invasion assays

Cell migration and invasion abilities were evaluated by performing transwell assay with the 24-well transwell chamber without (for migration assay) or with (for invasion assay) Matrigel (Becton Dickinson, San Jose, CA, USA). In brief, 1 × 10^5^ cells were inoculated into the top chamber of an insert with of serum-free medium (200 μL), while lower chamber was filled with medium with 10% FBS. After incubation for 48 h, the cells that on the upper surface of the membrane were removed, whereas the invaded or migrated cells were fixed in paraformaldehyde (4%), dyed with crystal violet (0.1%), and then imaged with a microscope (Olympus, Tokyo, Japan). The number of invaded and migrated cells was calculated in five randomly selected fields. Three independent experiments were conducted.

### Western blot assay

Cells were lysed in RIPA buffer (Solarbio) containing a proteinase/phosphatase inhibitors. Thirty micrograms of total protein was segregated by 10% or 12% SDS-PAGE, and then electrophoretically transferred onto PVDF membranes (Millipore, Billerica, MA, USA) at 280 mA for 1.5 h. These membranes were blocked in 5% non-fat milk, followed by incubation with the specific primary antibodies overnight at 4 °C. After that, the membranes were washed with TBST and then incubated with secondary antibody (ab150077; 1:1000 dilution; Abcam, Cambridge, MA, USA) for 2 h. Each band was detected and analyzed using an Clarity™ Western ECL Substrate Kit (Bio-Rad, Hercules, CA, USA) and Image Lab software 5.2 (Bio-Rad), respectively. Three independent experiments were conducted. The primary antibodies were listed as follows: E-cadherin (ab15148; 1:1000; Abcam), Vimentin (ab137321; 1:1000; Abcam), N-cadherin (ab18203; 1:1000; Abcam), Lactate dehydrogenase (LDHA; ab101562; 1:1000; Abcam), Hexokinase 2 (HK2; ab227198; 1:1000n; Abcam), Glucose transport protein type 1 (GLUT1; ab115730; 1:1000; Abcam), LARP1 (ab86359; 1:1000; Abcam), and β-actin (ab8227; 1:1000; Abcam).

### Glucose consumption and lactate production

Lung cancer cells were inoculated into 12-well plates. After transfection for 48 h, culture medium was collected for glucose and lactate concentration assay with glucose assay kit (Solarbio) and lactic acid assay kit (Solarbio), correspondingly. The relative of glucose consumption and lactate production were standardized to matched control groups. Three independent experiments were conducted.

### Dual-luciferase reporter assay

The candidate target miRNAs of circ_0047921 were predicted using Starbase 3.0 (http://starbase.sysu.edu.cn/) and circinteractome (https://circinteractome.nia.nih.gov/). The candidate target genes of miR-1287-5p were predicted using Starbase 3.0, TargetScanHuman (http://www.targetscan.org/), and miRDB (http://mirdb.org/). MiR-1287-5p binding sites of circ_0047921 or 3′untranslated region (UTR) of LARP1 were predicted using the bioinformatics databases. The sequences containing predicted miR-1287-5p interacting sites were respectively synthesized and inserted into the pGL3-basic vectors (Realgene, Nanjing, China), namely circ_0047921-WT and LARP1 3′UTR-WT, with mutant type luciferase reporter vectors (circ_0047921-MUT or LARP1 3′UTR-MUT) as controls. The lung cancer cells were co-transfected with indicated luciferase reporter vectors and miR-1287-5p mimic or miR-NC. After 48 h, luciferase activity was detected by the dual-luciferase reporter assay system (Promega, Madison, WI, USA) referring to the manufacturer’s procedures. Three independent experiments were conducted.

### In vivo experiment

Animal studies were approved by the Institutional Animal Care and Use Committee of The First People’s Hospital of Chenzhou, Southern Medical University. The athymic BALB/c nude mice (6–8 weeks; Vital River, Beijing, China) were used for animal studies and randomly divided into 2 groups (*N* = 6 per group). A549 cells infected with lentivirus-packaged sh-circ_0047921 (Genscript) were hypodermically inoculated into flank area of nude mice to cause xenografts (1 × 10^7^ cells/per mice) in pathogen-free conditions, with sh-NC as control. The volume of the xenografts was periodically examined based on formula: *V* (mm^3^) = 1/2 × *ab*^2^ (length (*a*) and width (*b*) length of the tumor). Thirty-five days after injection, mice were sacrificed and the tumors were excised for further analysis.

### Statistical analysis

All statistical analyses were conducted using SPSS 21.0 software (IBM, Somers, NY, USA) and data were exhibited as mean ± standard deviation. The statistically significant of measurement data between the two groups or more groups were conducted using Student’s *t* test or analysis of variance, respectively. *P* < 0.05 was considered statistically significant in all tests.

## Results

### Circ_0047921 was upregulated in lung cancer tissues and cells

**Fig. 1:**
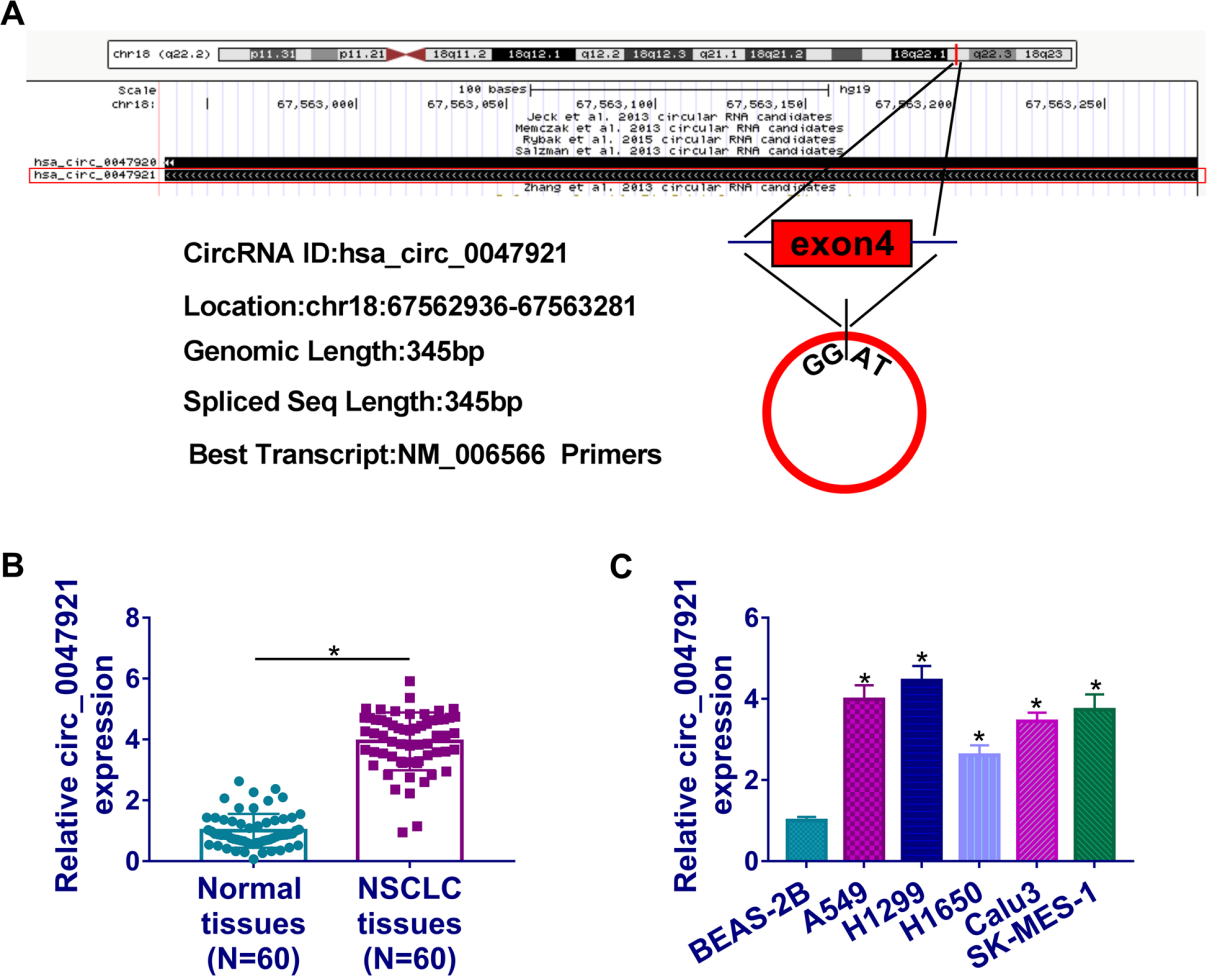
The expression level of circ_0047921 in lung cancer tissues and cells. **A** The information of circ_0047921 was presented. **B**, **C** The relative expression level of circ_0047921 was quantified by RT-qPCR in lung cancer tissues (NSCLC, *N* = 60) and neighboring normal tissues (*N* = 60), as well as in BEAS-2B and lung cancer cells. *P < 0.05

As presented in Fig. 1A, circ_0047921 was derived from exon4 of CD266 gene and located on chr18 (67592936-67563281). Besides, circ_0047921 expression level in lung cancer tissues was higher than that in matched normal lung tissues (Fig. 1B). Analogously, circ_0047921 was overexpressed in lung cancer cells compared with control BEAS-2B cells, especially in A549 and H1299 cells (Fig. 1C). These data suggested that circ_0047921 was upregulated in lung cancer tissues and cells.

### Circ_0047921 knockdown inhibited the proliferation, migration, invasion, EMT, and glycolysis of lung cancer cells

**Fig. 2:**
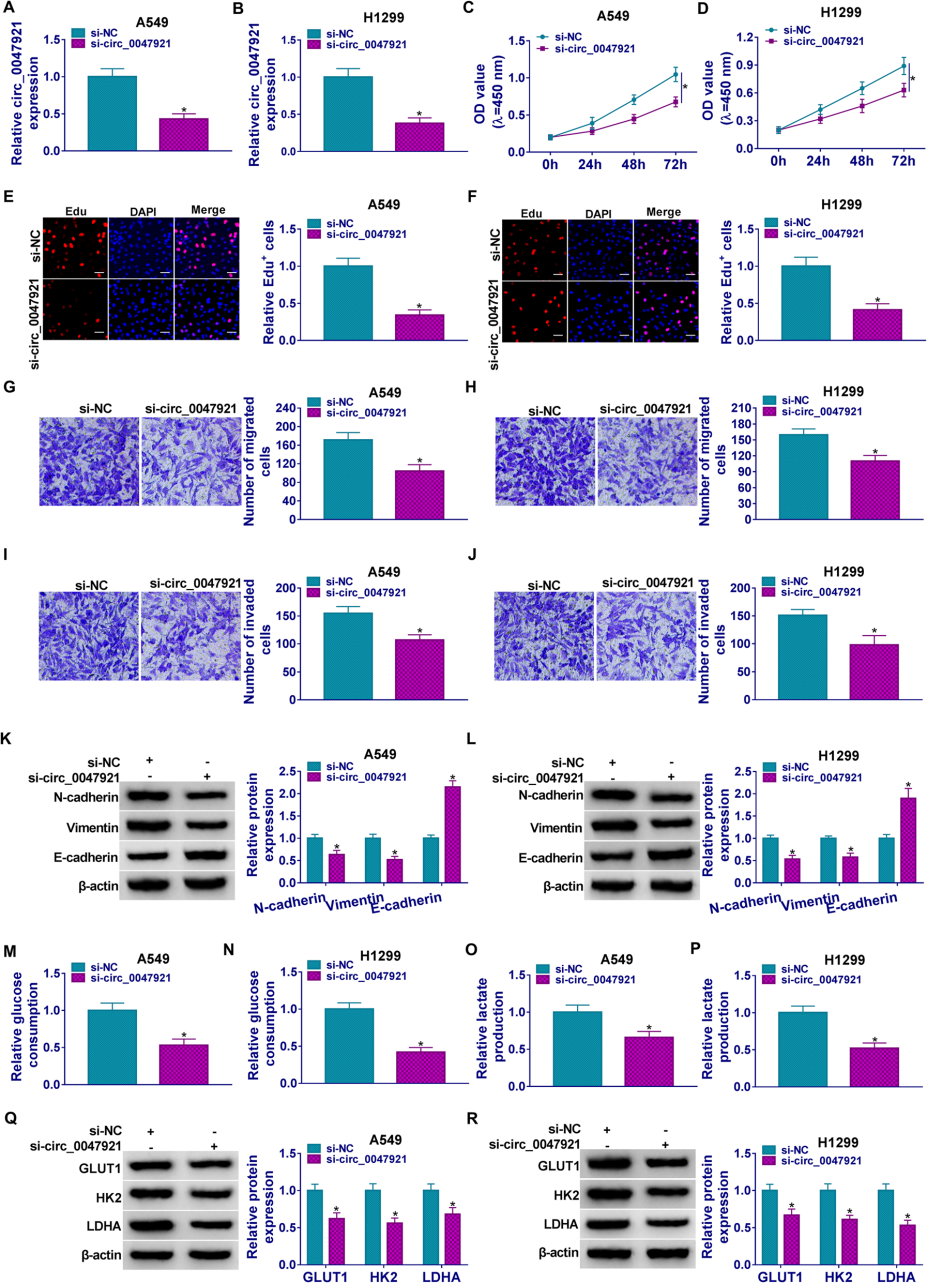
Effects of circ_0047921 inhibition on proliferation, migration, invasion, and EMT of lung cancer cells. **A**–**R** A549 and H1299 cells were transfected with si-NC or si-circ_0047921. **A**, **B** The relative expression level of circ_0047921 was evaluated by RT-qPCR. **C**, **D** The growth curves of A549 and H1299 cells were measured by CCK8 assay. **E**, **F** Enumeration of Edu-positive cells was evaluated by Edu assay. **G**, **J** Transwell assay was performed to measure the migration and invasion abilities of A549 and H1299 cells. **K**, **L** Western blot analysis was carried out to show E-cadherin, Vimentin and N-cadherin levels in A549 and H1299 cells. **M**, **P** The glucose consumption and lactate production were determined by matched kits. **Q**, **R** GLUT1, HK2, and LDHA levels in A549 and H1299 cells were quantified by western blot. *P < 0.05

The role of circ_0047921 inhibition was explored in A549 and H1299 cells. We found that circ_0047921 expression was greatly decreased in A549 and H1299 cells by si-circ_0047921 transfection (Fig. 2A, B). Subsequently, cell proliferation was inhibited in A549 and H1299 cells after circ_0047921 knockdown (Fig. 2C, D). Similarly, the knockdown of circ_0047921 reduced the number of EdU-positive cells, suggesting proliferation inhibition in A549 and H1299 cells (Fig. 2E, F). In addition, circ_0047921 silencing clearly reinforced migration and invasion of A549 and H1299 cells (Fig. 2G–J). The results of western blot analysis showed that circ_0047921 suppression triggered a remarkable suppressive effect on EMT process by decreasing Vimentin and N-cadherin, and increasing E-cadherin in A549 and H1299 cells (Fig. 2K, L). Transfection with si-circ_0047921 prominently constrained glycolysis by reducing glucose consumption and lactate production in A549 and H1299 cells (Fig. 2M–P). Consistently, LDHA, HK2, and GLUT1 were all downregulated in A549 and H1299 cells after transfection with si-circ_0047921 (Fig. 2Q, R). Collectively, circ_0047921 exerted a tumor facilitator role in lung cancer progression.

### Circ_0047921 targeted miR-1287-5p in lung cancer cells

**Fig. 3:**
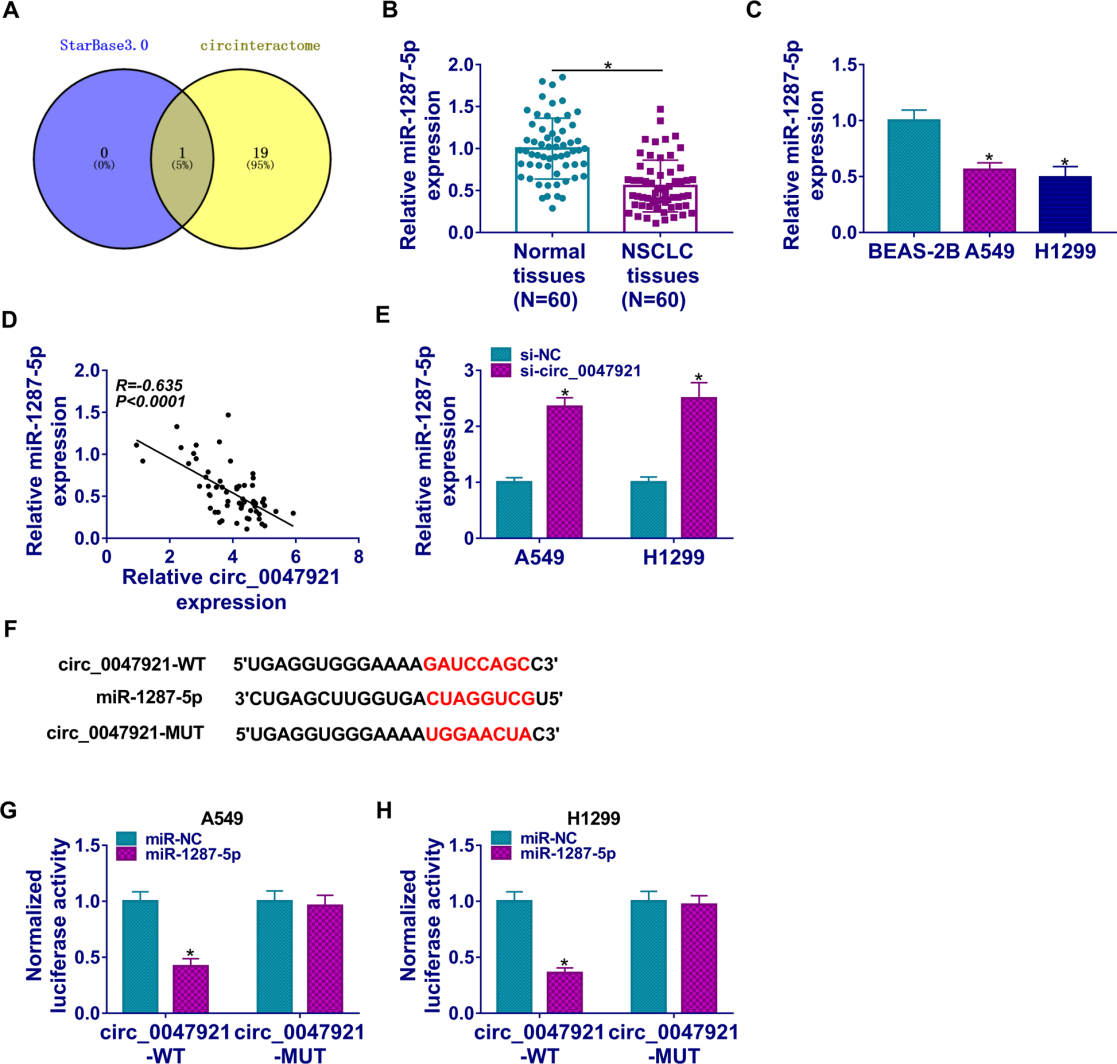
MiR-1287-5p was a direct target of circ_0047921. **A** The putative targets of circ_0047921 were predicted by Starbase 3.0 and circinteractome. **B**, **C** RT-qPCR assay was used to detect the expression level of miR-1287-5p in lung cancer tissues (NSCLC, *N* = 60) and corresponding controls (*N* = 60), as well as in BEAS-2B, A549, and H1299 cells. **D** Correlation analysis revealed a negative correlation between miR-1287-5p and circ_0047921 in tumor tissues. **E** The expression level of miR-1287-5p was examined by RT-qPCR assay in A549 and H1299 cells infected with si-NC or si-circ_0047921. **F** Binding regions between miR-1287-5p and circ_0047921 were shown. **G**, **H** The relative luciferase activity in A549 and H1299 cells was analyzed by dual-luciferase reporter assay. *P < 0.05

In order to know the direct targets of circ_0047921, we performed bioinformatics analysis. Based on the combined prediction of Starbase 3.0 and circinteractome, only miR-1287-5p was screened out as a candidate (Fig. 3A). The data suggested that miR-1287-5p harbored binding sites on circ_0047921 (Fig. 3A). Importantly, miR-1287-5p was decreased in lung cancer tissues and cells than that in matched control groups (Fig. 3B, C). In addition, a negative correlation between circ_0047921 and miR-1287-5p expression was identified in lung cancer tissues (Fig. 3D). We also observed that miR-1287-5p level was increased in A549 and H1299 cells transfected with si-circ_0047921 contrasted to si-NC (Fig. 3E). The binding sequences of miR-1287-5p to circ_0047921 were presented in Fig. 3F. Furthermore, dual-luciferase reporter assay results indicated that luciferase activity of circ_0047921-WT reporter was apparently inhibited by miR-1287-5p overexpression, while the circ_0047921-MUT reporter was not changed by miR-1287-5p (Fig. 3G, H). In summary, miR-1287-5p was a target of circ_0047921 in lung cancer.

### Inhibition of miR-1287-5p reversed the effects of circ_0047921 silencing on proliferation, migration, invasion, EMT, and glycolysis of lung cancer cells

**Fig. 4:**
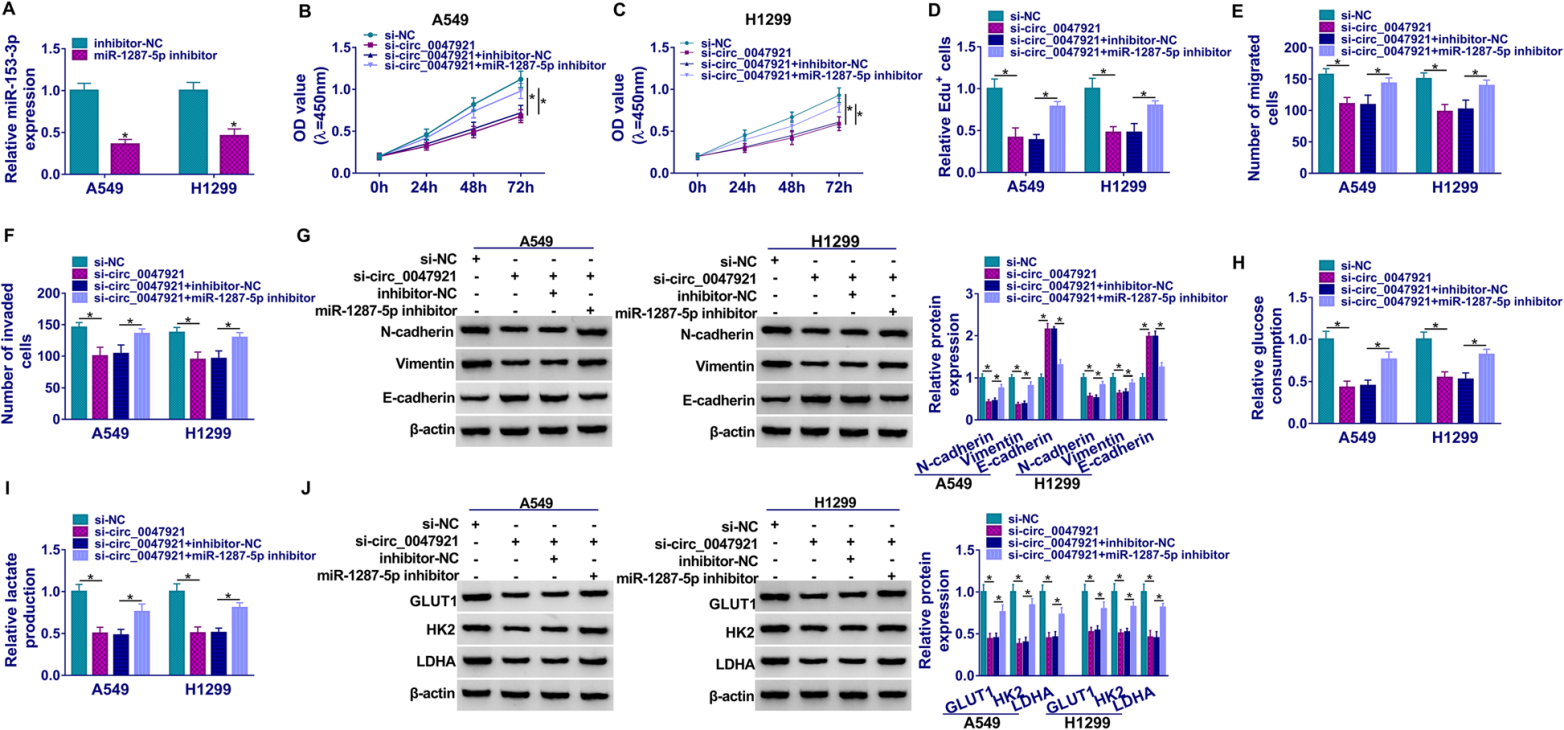
Silencing of circ_0047921-mediated effects on proliferation, migration, invasion, EMT and glycolysis of lung cancer cells could be reversed by knockdown of miR-1287-5p. **A** The expression of miR-1287-5p was assessed with RT-qPCR in A549 and H1299 cells transfected with inhibitor-NC or miR-1287-5p inhibitor. **B**–**J** A549 and H1299 cells were transfected with si-NC, si-circ_0047921, si-circ_0047921 + inhibitor-NC, or si-circ_0047921 + miR-1287-5p inhibitor. **B**, **C** The cell viability was assessed with CCK8 assay. **D** Edu assay was performed in A549 and H1299 cells. **E**, **F** Transwell assay was used to analyze migration and invasion abilities of transfected A549 and H1299 cells. **G** The western blot assay was applied to test E-cadherin, Vimentin, and N-cadherin expression. **H**, **I** Glucose consumption and lactate production of transfected A549 and H1299 cells were displayed. **J** Expression of LDHA, HK2, and GLUT1 was assessed by western blot assay in A549 and H1299 cells. *P < 0.05

MiR-1287-5p expression was greatly repressed in A549 and H1299 cells transfected with miR-1287-5p inhibitor when in comparison with the inhibitor-NC transfected cells (Fig. 4A). Moreover, silencing of miR-1287-5p could partly rescue the cell viability loss in A549 and H1299 cells caused by knockdown of circ_0047921 (Fig. 4B–D). Co-transfection of si-circ_0047921 and miR-1287-5p inhibitor could counteract the inhibitory effects on migration and invasion in A549 and H1299 cells induced by si-circ_0047921 (Fig. 4E, F). Additionally, transfection of si-circ_0047921 impeded EMT progression by regulation of E-cadherin, Vimentin, and N-cadherin expression, which was overturned by silencing of miR-1287-5p (Fig. 4G). Similarly, downregulation of circ_0047921 impeded glycolysis, which could be abated by transfection with miR-1287-5p inhibitor (Fig. 4H, I). Finally, result of western blot revealed that LDHA, HK2, and GLUT1 expression was reduced by si-circ_0047921 but rescued by miR-1287-5p inhibitor (Fig. 4J). Altogether, these data implicated that miR-1287-5p was a target of circ_0047921.

### LARP1 was upregulated in lung cancer tissues and cells and was a target gene of miR-1287-5p

**Fig. 5:**
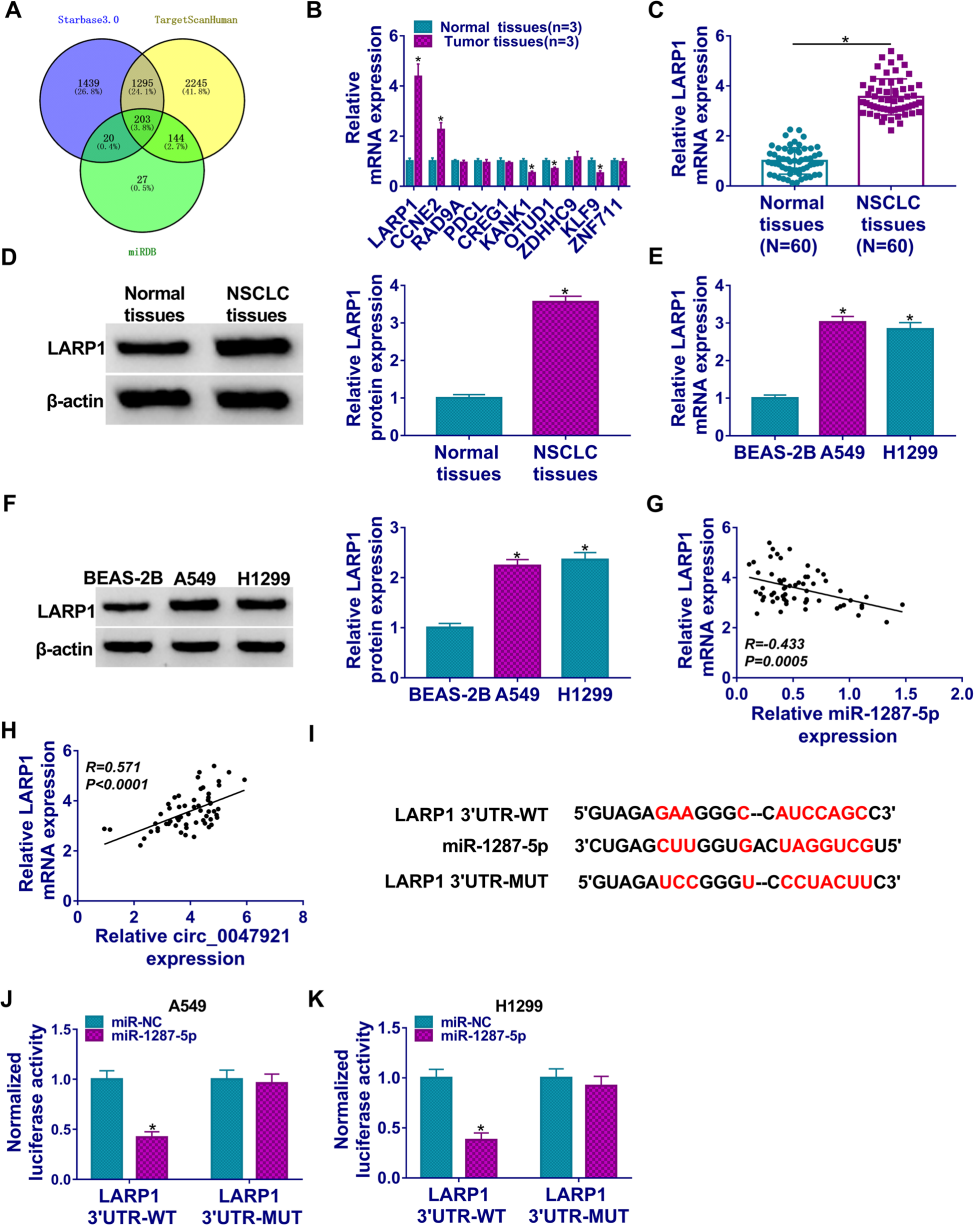
LARP1 was a target of miR-1287-5p in lung cancer. **A** The putative binding targets of miR-1287-5p were predicted by miRDB, TargetScanHuman, and Starbase 3.0. **B** The expression levels of 10 selected mRNAs were detected by RT-qPCR assay in lung tumor tissues (*n* = 3) and normal lung tissues (*n* = 3). **C**–**F** The expression level of LARP1 was assessed by RT-qPCR and western blot assays in lung cancer tissues (*N* = 60) and cells. **G**, **H** The relationship between LARP1 and miR-1287-5p or circ_0047921 was analyzed in lung cancer tissues. **I** Binding regions between LARP1 and miR-1287-5p were shown. **J**, **K** Dual-luciferase reporter assay was conducted to assess the luciferase activity in A549 and H1299 cells co-transfected with LARP1 3′UTR-WT/MUT and miR-1287-5p/miR-NC. *P < 0.05

Through the common prediction of miRDB, TargeScan, and Starbase 3.0, we screened out the potential targeting genes of miR-1287-5p. A total of 203 mRNAs were found in the intersection part (Fig. 5A). Then we selected 10 mRNAs to detect their expression in lung tumor tissues. The data showed that LARP1 level was significantly increased in lung cancer tissues (Fig. 5B). In addition, the results of RT-qPCR and western blot assay also suggested that LARP1 levels were upregulated in lung cancer tissues and cells than that in adjacent non-tumorous tissues and BEAS-2B cells, respectively (Fig. 5C–F). Furthermore, LARP1 was negatively correlated with miR-1287-5p while positively correlated circ_0047921 expression in lung cancer tissues (Fig. 5G, H). As shown in Fig. 5I, miR-1287-5p possessed the complementary sequences to 3′UTR of LARP1. We carried out dual-luciferase reporter assay which indicated that miR-1287-5p directly bound to 3′UTR of LARP1 in both A549 and H1299 cells (Fig. 5J, K). On the whole, these data implicated that LARP1 was a target of miR-1287-5p in lung cancer.

### Silencing of circ_0047921-mediated suppression effects on proliferation, migration, invasion, EMT, and glycolysis of lung cancer cells were abolished by overexpression of LARP1

**Fig. 6:**
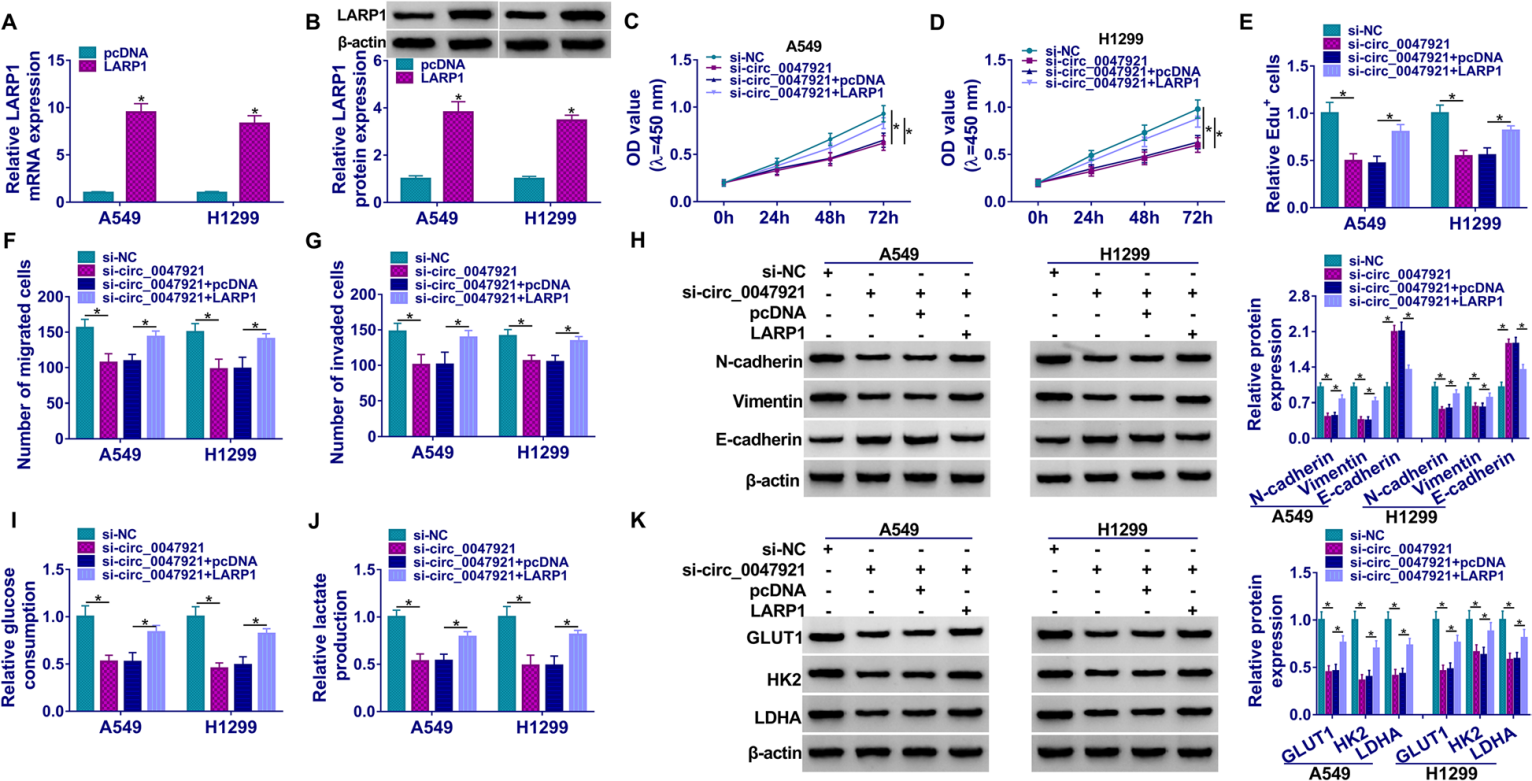
Overexpression of LARP1 could abolish silencing of circ_0047921-mediated effects on proliferation, migration, invasion, EMT and glycolysis of lung cancer cells. **A**, **B** RT-qPCR and western blot assays were conducted to confirm the overexpression efficiency of LARP1 in A549 and H1299 cells. **C**–**K** A549 and H1299 cells were transfected with si-NC, si-circ_0047921, si-circ_0047921 + vector, or si-circ_0047921 + LARP1. **C**, **D** CCK8 and Edu assays were conducted for measuring the cell viability and Edu-positive cells. **F**, **G** Transwell assay was conducted to measure migration and invasion in transfected A549 and H1299 cells. **H** The protein expressions levels of E-cadherin, Vimentin, and N-cadherin were quantified by western blot assay. **I**, **J** Glucose consumption and lactate production of transfected A549 and H1299 cells were presented. **K** Expression of LDHA, HK2, and GLUT1 was calculated by western blot assay. *P < 0.05

We measured the mRNA and protein levels of LARP1 in A549 and H1299 cells. As presented in Fig. 6A, B, transfection with LARP1 markedly upregulated the LARP1 level in A549 and H1299 cells. Besides, we found that transfection with si-circ_0047921 could decrease proliferation, while this inhibition was abolished by transfecting with LARP1 (Fig. 6C–E). Importantly, overexpression of LARP1 rescued migration and invasion inhibition in A549 and H1299 cells induced by circ_0047921 knockdown (Fig. 6F, G). The results of western blot assay showed that overexpression of LARP1 strikingly abolished the suppression effects on EMT process in A549 and H1299 cell induced by knockdown of circ_0047921 (Fig. 6H). Furthermore, knockdown of circ_0047921 inhibited cell glycolysis, whereas this action was weakened by upregulation of LARP1 (Fig. 6I, J). Silencing of circ_0047921 significantly decreased LDHA, HK2, and GLUT1 expression, but it was rescued by LARP1 overexpression (Fig. 6K). Therefore, circ_0047921 mediated LARP1 expression to promote lung cancer progression.

### Circ_0047921 regulated LARP1 expression by miR-1287-5p

**Fig. 7:**
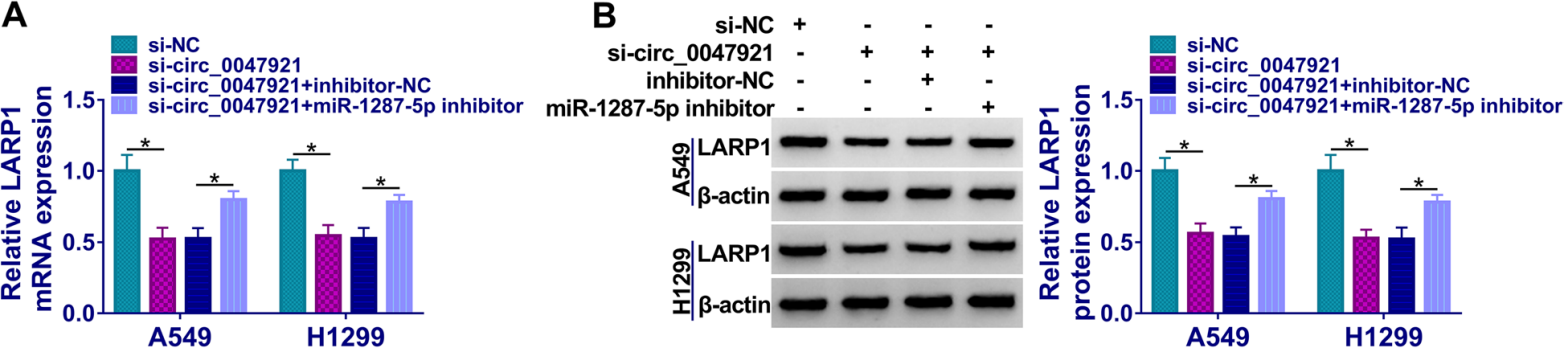
Circ_0047921 regulated LARP1 by sponging miR-1287-5p. **A**, **B** The protein expression levels of LARP1 were evaluated by RT-qPCR and western blot assays in A549 and H1299 cells transfected with si-NC, si-circ_0047921, si-circ_0047921 + inhibitor-NC, or si-circ_0047921 + miR-1287-5p inhibitor. *P < 0.05

As show in Fig. 7A, B, downregulation of circ_0047921 suppressed the LARP1 expression in A549 and H1299 cells, and silencing of miR-1287-5p reversed the si-circ_0047921-induced effects, hinting that circ_0047921/miR-1287-5p/LARP1 axis played significant role in lung cancer progression.

### Circ_0047921 silencing impeded the growth of tumor in vivo

**Fig. 8:**
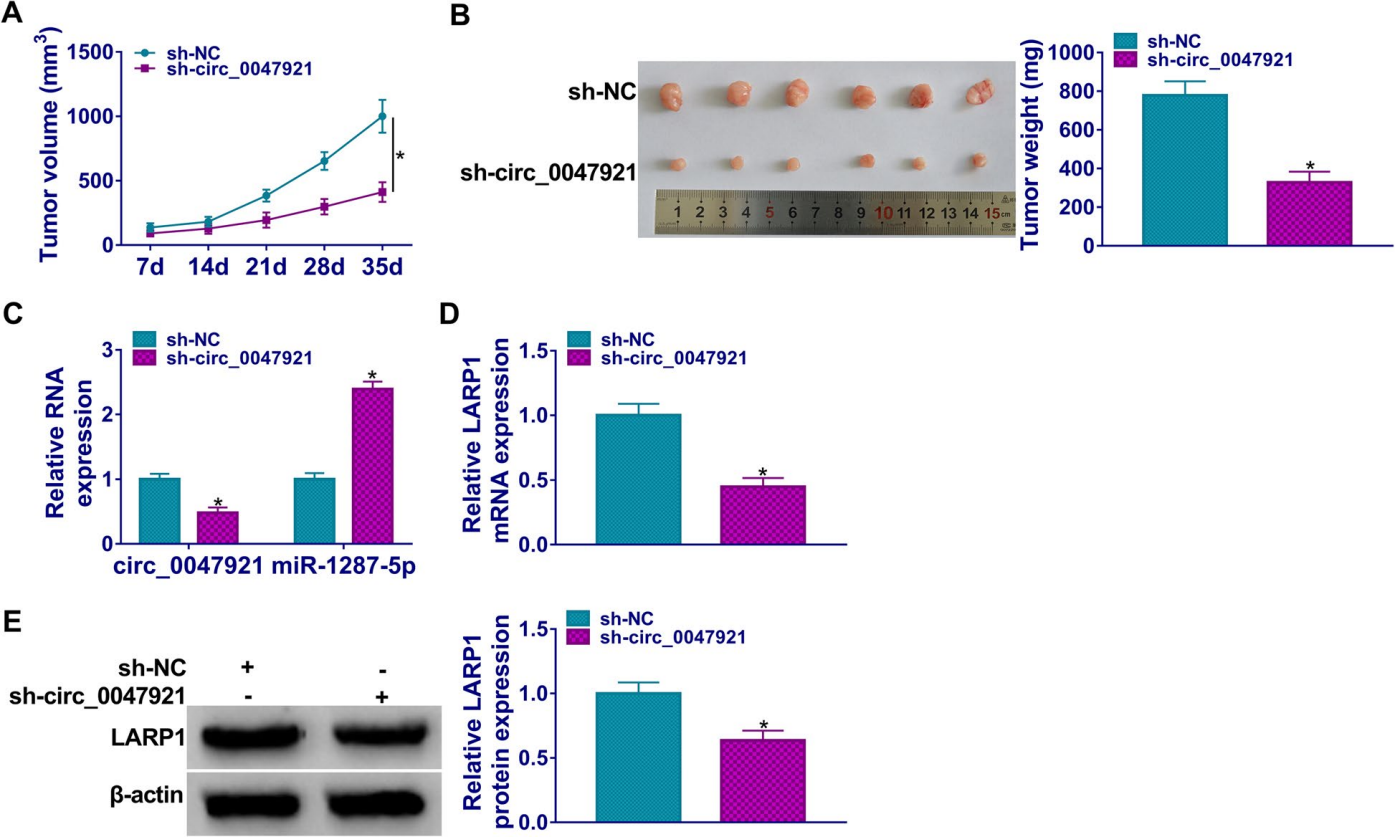
Impact of circ_0047921 inhibition on tumor growth in vivo. **A**, **B** The growth curves and weight of xenograft tumors were shown. **C**–**E** The expression levels of circ_0047921, miR-1287-5p, and LARP1 (mRNA and protein) were estimated with RT-qPCR and western blot assays. *P < 0.05

The functional effects of circ_0047921 were explored on tumor growth *in vivo*. The downregulation of circ_0047921 markedly decreased the growth of the tumors and weight compared with control (Fig. 8A, B). As expected, circ_0047921 and LARP1 expression were decreased, while miR-1287-5p expression was increased in sh-circ_0047921 group compared with sh-NC group (Fig. 8C–E). Collectively, circ_0047921 silencing impeded lung cancer development in vivo.

## Discussion

Currently, we identified that circ_0047921 expression was overly increased in lung cancer tissues and cells. Circ_0047921 inhibition impeded lung cancer progression by regulation of cell proliferation, migration, invasion, EMT, and glycolysis. Conclusively, circ_0047921 regulated the development of lung cancer through regulating miR-1287-5p/LARP1 axis.

The competing endogenous RNA (ceRNA) hypothesis was validated and attracted great attention in many different biological events [10]. A previous study clarified that circ_0047921, circ_0007761, and circ_0056285 might be diagnostic targets of NSCLC [21]. However, their biological effects were unknown. Thus, the inquiry on whether circ_0047921 functioned as a ceRNA was meaningful. In this study, we found that circ_0047921 knockdown inhibited lung cancer cell proliferation, migration, invasion, EMT, and glycolysis. EMT and glycolysis were closely associated with tumorigenesis [22, 23], indicating that circ_0047921 knockdown blocked lung cancer development. Moreover, we demonstrated that circ_0047921 acted as a ceRNA of miR-200a-3p in lung cancer cells.

Recent researches have revealed that deregulation of miRNAs was associated with pathogenesis of lung cancer by mediating cell growth, apoptosis, and metastasis [24, 25]. Ji et al. declared that miR-1287-5p acted a tumorigenic role in cervical cancer by inhibiting proliferation and tumor growth [26]. The anti-tumorigenic function of miR-1287-5p contributed to regulate oncogene expression in different diverse of tumors [27]. Similar with previous conclusions [14, 15], miR-1287-5p acted as a tumor inhibitor miRNA in lung cancer. Moreover, the regulatory mechanism of miR-1287-5p in EMT and glycolysis was scarcely documented. Currently, miR-1287-5p was significantly downregulated in lung cancer tissues and cells, and miR-1287-5p inhibition counteracted the inhibiting effects of si-circ_0047921 on lung cancer cell growth, migration, invasion, EMT, and glycolysis, indicating that circ_0047921 regulated circ_0047921 progression by targeting miR-1287-5p.

Additionally, LARP1 was a functional target of miR-1287-5p and was inversely related to miR-1287-5p expression in lung cancer tissues. The abnormal expression of LARP1 was highly related to tumor occurrence and metastasis [28]. Analogously, Han et al. also revealed that LARP1 promoted lung cancer progression by regulating growth and mobility of cancer cells [29]. Besides, Hopkins et al. pointed out that LARP1 was compulsory for cancer cell proliferation and drug-resistance and promoted tumor formation and maintained cancer stem cell-like populations [30]. It was noteworthy that LARP1 was recently reported to promote lung development through miRNAs-driven, including miR-503 and miR-374a [19, 29]. Not surprisingly, the cancerogenic role of LARP1 was also confirmed in our research depending on miR-1287-5p regulation. The upregulation of LARP1 could abolish inhibitory effects of circ_0047921 silencing on lung cancer progression.

In summary, our current work implied that the carcinogenic role of circ_0047921 was partly attributed to its regulatory effects on miR-1287-5p/LARP1 axis, and circ_0047921 might serve as a novel biomarker and a promising therapeutic target for lung cancer patients.

## Conclusion

Collectively, in this study we firstly confirmed that circ_0047921/miR-1287-5p/LARP1 axis facilitated the development of lung cancer by regulating growth, migration, invasion, EMT and glycolysis of lung cancer cells. Mechanically, circ_0047921 sponged miR-1287-5p to increase LARP1 expression, thereby aggravating the development of lung cancer.

## Data Availability

The analyzed data sets generated during the present study are available from the corresponding author on reasonable request.
